# Modern Treatment of Skeletal Metastases: Multidisciplinarity and the Concept of Oligometastasis in the Recent Literature

**DOI:** 10.3390/curroncol32040226

**Published:** 2025-04-11

**Authors:** Giulia Trovarelli, Arianna Rizzo, Felicia Deborah Zinnarello, Mariachiara Cerchiaro, Andrea Angelini, Elisa Pala, Pietro Ruggieri

**Affiliations:** 1 Department of Orthopedics and Orthopedic Oncology, University of Padua, 35122 Padua, Italy; giulia.trovarelli@unipd.it (G.T.); feliciadeborah.zinnarello@aopd.veneto (F.D.Z.); mariachiara.cerchiaro@unipd.it (M.C.); andrea.angelini@unipd.it (A.A.); elisa.pala@unipd.it (E.P.); 2 Department of Surgery, Oncology and Gastroenterology (DISCOG), University of Padova, 35122 Padua, Italy; 3 Department of Medical and Surgical Specialties, Radiological Sciences and Public Health, University of Brescia, Viale Europa 11, 25123 Brescia, Italy; ariannarizzo22@gmail.com

**Keywords:** bone metastases, surgical treatment, oligometastases, multidisciplinary approach, palliative care, quality of life

## Abstract

Bone metastases are a major concern in cancer management since they significantly contribute to morbidity and mortality. Metastatic lesions, commonly arising from breast, prostate, lung, and kidney cancers, affect approximately 25% of cancer patients, leading to severe complications such as pain, fractures, and neurological deficits. This narrative review explores contemporary approaches to bone metastases, emphasizing a multidisciplinary strategy and the evolving concept of oligometastatic disease. Oligometastases, defined by limited metastatic spread (1–5 lesions), offer a potential window for curative treatment through aggressive interventions, including stereotactic ablative radiotherapy and resection surgery. Tumor boards, integrating systemic therapies with local interventions, are crucial to optimize treatment. Despite promising results, gaps remain in defining optimal treatment sequences and refining patient selection criteria. Future research should focus on personalized approaches, leveraging biomarkers and advanced imaging to enhance outcomes and the quality of life in patients with bone metastases.

## 1. Introduction

Bone metastases are a significant concern in cancer care as they impact a substantial number of patients and contribute heavily to cancer-related mortality and morbidity. Studies suggest that metastatic disease accounts for over 90% of cancer-related deaths, with bone being a prevalent site for secondary lesions, especially in cancers of the breast, prostate, lung, and kidney [1,2,3,4]. Approximately one in up to four cancer patients will develop bone metastases during their illness [5,6,7], highlighting the critical need for effective management strategies.

The pathophysiology of bone metastasis involves a complex interplay between metastatic tumor cells and the bone microenvironment, often resulting in osteolytic or osteoblastic lesions, depending on the cancer type [8]. Breast and lung cancers typically induce osteolytic lesions by promoting increased osteoclast activation, leading to bone destruction [9]. In contrast, prostate cancer often results in osteoblastic lesions due to abnormal bone formation [8,10]. Both types of metastases lead to severe complications, such as pain, fractures, hypercalcemia, and neurological deficits in cases of spinal involvement [11,12,13]. Such complications significantly deteriorate quality of life and functional capacity, stressing the need for integrated treatment approaches that not only alleviate symptoms but also aim to maintain structural stability and mobility.

The management of bone metastases has increasingly adopted a multidisciplinary approach, integrating systemic, radiotherapeutic, and surgical strategies [14,15,16]. Based on biomarkers and histological analyses, a more personalized treatment approach is possible and should serve as the foundation for guiding these interventions. Chemotherapy and radiotherapy remain the cornerstone treatments for patients with skeletal metastases. However, surgery is still essential to relieve symptoms, restore function, and prevent skeletal-related events (SREs), such as fractures, which can severely impact quality of life and mobility [17,18,19]. Recent studies [20] have reported that curative surgery is possible for oligometastatic patients and those with solitary lesions [21,22]. However, data on long-term outcomes and post-surgical quality of life are limited.

This review aims to recap current findings on modern treatments for bone metastases, emphasizing the importance of a multidisciplinary approach and the concept of oligometastases as crucial factors in improving patient outcomes.

## 2. Materials and Methods

This narrative review outlines the fundamental principles for treating metastatic bone disease by collecting articles published in English from various countries, including reviews, clinical trials, and meta-analyses. The literature sources utilized were PubMed, Google Scholar, ClinicalTrials.gov, and relevant academic conferences. The search strategy used for the literature review was metast* AND oligomet* AND (bone OR skelet*) AND (treatment OR radiot* OR chemo* OR surg*) AND multidisciplin* AND (team OR approach OR treatment OR care). A total of 1064 articles were reviewed. The articles chosen were published between 2000 and 2024, with the search strategy detailed in each section. Papers deemed less reliable or those that more recent studies with similar content have surpassed were excluded. Most of the literature was selected by author GT, with additional contributions by AR and DZ, and reviewed by all authors. The selected articles were synthesized and analyzed, incorporating the authors’ perspectives and insight.

## 3. Discussion

### 3.1. Oligometastatic Disease

Hellman and Weichselbaum introduced oligometastases in 1995 [23] to describe an intermediate state between localized and widely metastatic disease that could potentially be amenable to curative treatment. This concept links oligometastatic disease to limited metastatic spread, typically consisting of 1–5 lesions within a single anatomical region, and associates disease-free survival with favorable prognostic outcomes [20,24]. In simpler terms, oligometastases refer to a state where cancer has spread to a limited number of sites, making it potentially curable [21,22].

In 2020, an international panel of experts from EORTC and ESTRO proposed a consensus framework for better characterizing and classifying metastatic disease [25]. This framework, a significant development in the field, categorizes oligometastases into three types: de novo oligometastatic, induced oligometastatic, and repeat oligometastatic. De novo oligometastatic disease refers to newly diagnosed metastases, which can be classified as synchronous if detected within six months of the primary tumor diagnosis or metachronous if diagnosed more than six months after the primary tumor. Induced oligometastatic disease arises when systemic therapy reduces the number of metastases. Finally, repeat oligometastatic disease refers to recurrence in a previously treated patient with controlled primary tumors and stable disease. These classifications also incorporate the response of metastatic lesions to systemic therapy.

Recent clinical trial results [26,27] and the evolving understanding of metastatic disease have expanded the definition of oligometastasis. This broader definition includes cases with less favorable prognoses due to histological subtype, metastatic burden, and patient-specific risk factors. The oligometastatic state offers a unique window for potentially curative interventions. Prognostic models have been developed to predict outcomes based on tumor type, metastatic distribution, and therapeutic approaches. Patients with oligometastatic disease can take advantage of the potential benefits of combining local and systemic treatments [21,22]. Aggressive local interventions, such as surgical resection or stereotactic ablative radiotherapy (SABR), have improved overall survival and quality of life.

Recognizing and effectively managing oligometastatic disease is paramount for optimizing patient outcomes, particularly in skeletal metastases, where targeted therapies can offer significant benefits. As a result, more aggressive treatment strategies, including surgery and SABR, are being increasingly applied even in cases previously considered unsuitable for local therapy. For example, Gomez et al. [28,29] showed that local consolidative therapies significantly enhanced progression-free survival in patients with oligometastatic non-small cell lung cancer.

### 3.2. Medical Therapy

Systemic treatments are crucial in managing metastatic bone disease [30,31] since they target primary and micrometastatic diseases to control tumor progression and symptoms [25], aiming for prolonged survival and improved quality of life [18].

Besides conventional chemotherapy agents, bone-modifying agents and new systemic treatments are now available, and they are usually used in patients with bone metastases (Table 1).

Bisphosphonates are the primary bone-modifying agents used in clinical practice [32,33,34,35,36,37,38,39,40]. They inhibit bone resorption by interfering with osteoclast function thanks to their binding to hydroxyapatite crystals and incorporation into the bone matrix. Additionally, they help limit the release of growth factors that promote tumor progression in the bone microenvironment [41]. As a result, they reduce SREs, control pain, and improve quality of life [42]. The most commonly used bisphosphonates (zoledronic acid, pamidronate, and ibandronate) are employed in various cancers, including breast, prostate, lung, and multiple myeloma [34,35,36,43]. However, their use is associated with renal toxicity and osteonecrosis of the jaw [9,44,45]. More recently, Denosumab, a monoclonal antibody, was introduced as an alternative bone-modifying agent. It inhibits osteoclast-mediated bone resorption by binding to RANKL (Receptor Activator of Nuclear Factor Kappa-B Ligand), preventing its interaction with osteoclast receptors. This mechanism reduces osteoclast attachment, induces apoptosis, and decreases bone degradation [44,45]. Denosumab has demonstrated efficacy comparable to bisphosphonates in preventing SREs in patients with bone metastases [46]. Its advantages include subcutaneous administration and a lower risk of renal toxicity, making it a preferred option for patients with pre-existing renal impairment [39]. Bone-modifying agents are most effective when combined with systemic therapies and local treatments such as radiation or surgery. However, their potential role in preventing new bone metastases remains under investigation. Coleman et al. [47] studied the role of adjuvant bisphosphonates in reducing bone metastases and improving survival in postmenopausal women with early breast cancer. A key biomarker in predicting the response to bisphosphonates is MAF (v-Maf Avian Musculoaponeurotic Fibrosarcoma oncogene homolog): patients with MAF-negative tumors benefit from treatment, whereas those with MAF-positive tumors, particularly in the presence of circulating estrogens, may experience worse outcomes due to an increased risk of extra-skeletal metastases. The Early Breast Cancer Trialists’ Collaborative Group confirmed through meta-analysis that these protective effects are primarily observed in postmenopausal patients, likely due to hormonal influences [48]. Current guidelines recommend bisphosphonates for postmenopausal women at high risk of recurrence, and MAF testing may help refine patient selection.

New systemic treatments include hormone therapy, targeted therapy, and immunotherapy, depending on the type of tumor.

Hormone therapy [49,50,51,52,53,54,55,56,57,58,59,60,61] is a cornerstone in the treatment of hormone-sensitive metastatic cancers, such as breast and prostate cancer, since it blocks the effects of those hormones on cancer growth and bone metastases development [18,62].

Estrogen receptor-positive (ER-positive) breast cancer cells depend on estrogen for growth and proliferation. Aromatase inhibitors (AIs), such as letrozole, anastrozole, and exemestane, inhibit the enzyme aromatase, which converts androgens into estrogens in peripheral tissues. These agents are widely used in postmenopausal women, where circulating estrogens derive mainly from peripheral conversion rather than ovarian production [51,54]. Tamoxifen, a selective estrogen receptor modulator (SERM), binds to estrogen receptors on cancer cells, blocking estrogen’s ability to stimulate tumor growth. It is commonly used in premenopausal women [57] in combination with ovarian suppression or ablation (via surgery or luteinizing hormone-releasing hormone (LHRH) agonists such as goserelin) to reduce further circulating estrogen levels [53].

On the other hand, prostate cancer cells rely on androgens for growth and survival. Therefore, androgen deprivation therapy (ADT) is the cornerstone of treatment for metastatic prostate cancer, particularly in patients with bone metastases. LHRH agonists, such as leuprolide and goserelin, suppress testosterone production by the testes, leading to a dramatic reduction in circulating androgen levels. Anti-androgens, such as bicalutamide, flutamide, and enzalutamide, are often used in combination with LHRH agonists to block the androgen receptor directly [49,60]. Additionally, abiraterone inhibits CYP17, an enzyme involved in androgen biosynthesis, thereby reducing androgen production from the adrenal glands, testes, and even the tumor [58].

Targeted therapies [63,64,65,66,67,68] inhibit specific proteins, enzymes, or molecules involved in cancer cell proliferation and spread; thus, they are more selective than traditional chemotherapy and often lead to fewer side effects [69]. Various targeted therapies have demonstrated promising results in reducing bone metastases and improving survival across different cancer types.

In Human Epidermal growth factor Receptor 2 (HER-2) positive breast cancer, monoclonal antibodies that inhibit HER2 (Trastuzumab) [67] or disrupt HER2 dimerization (Pertuzumab) [70] as well as antibody-drug conjugate that delivers chemotherapy directly to HER2-positive cancer cells (Ado-trastuzumab emtansine) [71], are commonly used for improving efficacy while reducing systemic toxicity. Additionally, inhibitors of cyclin-dependent kinases 4 and 6 (CDK4/6), such as Palbociclib, Ribociclib, and Abemaciclib, prevent cancer cell proliferation by blocking the cell cycle [72].

In prostate cancer, Abiraterone inhibits androgen biosynthesis, whereas Enzalutamide blocks androgen receptor activity [68]. Another practical approach is radium-223, a radiopharmaceutical that selectively targets bone metastases. By emitting high-energy alpha particles, radium-223 destroys cancer cells and limits its damage to surrounding healthy tissue [73]. Therapies targeting molecular abnormalities have also significantly improved patient outcomes in non-small cell lung cancer (NSCLC). Epidermal Growth Factor Receptor (EGFR) inhibitors, such as Erlotinib and Gefitinib, block the EGFR pathway, essential for cancer cell proliferation [64]. Additionally, Crizotinib, an anaplastic lymphoma kinase (ALK) inhibitor, explicitly targets ALK gene fusions found in a subset of NSCLC cases.

Other cancers also benefit from targeted therapies, including tyrosine kinase inhibitors (TKIs) like Sunitinib and Pazopanib, which inhibit vascular endothelial growth factor (VEGF) signaling. This pathway is critical for tumor angiogenesis and metastasis, making these drugs valuable in slowing disease progression [63].

Immunotherapy [18,47,74,75,76] harnesses immune response activation in the tumor microenvironment to target and destroy cancer cells using checkpoint inhibitors [69], such as those targeting programmed cell death protein-1 (PD-1) and its ligand (PD-L1). Pembrolizumab, Nivolumab (anti-PD-1), and Atezolizumab (anti-PD-L1) restore T-cell activity, enhancing immune response against bone metastatic cancers, including lung, renal, and melanoma [74]. However, the immunosuppressive bone environment, influenced by osteoclast activity and bone-derived growth factors, can limit the effectiveness of these therapies [76]. To solve this problem, clinical trials are currently exploring immune checkpoint inhibitors in combination with bone-modifying agents like bisphosphonates and Denosumab [47].

Chimeric antigen receptor (CAR) T-cell therapy, which involves genetically modifying the patient’s T cells to recognize and attack tumor-specific antigens, is an emerging approach in metastatic cancers, including multiple myeloma. The CAR-T therapy Idecabtagene Vicleucel (ide-cel) showed efficacy in patients with bone multiple myeloma [77].

Recently, several studies have explored the role of nanotechnology in cancer treatment, with a particular focus on breast cancer and its bone metastasis [78,79,80]. For example, Vanni et al. [78] investigated engineered anti-HER2 drug delivery nanosystems using PLGA nanoparticles loaded with trastuzumab to improve targeted therapy for HER2-positive breast cancer. Their findings demonstrated enhanced drug efficacy and localized release through a light-triggered system. However, despite advancements in nanoparticle-based drug delivery systems, challenges remain in achieving precise targeting of both tumor cells and the bone microenvironment, as well as addressing safety concerns [79,80]. Additionally, there are still significant obstacles in translating these strategies into clinical practice [80].

Despite the improvement in managing skeletal metastases guaranteed by these new treatments, challenges remain and are mainly related to the high heterogeneity of skeletal metastases with variable responses based on tumor type. Moreover, the development of resistance to targeted therapies and immunotherapies in bone metastases is a major hurdle in cancer treatment, often leading to treatment failure and disease progression. In bone metastases, cancer cells interact extensively with the bone microenvironment, creating a dynamic and complex ecosystem that contributes to therapeutic resistance. For targeted therapies, resistance can develop through several mechanisms, including mutations in the target proteins, activation of alternative signaling pathways, or upregulation of compensatory pathways that bypass the drug’s mechanism of action. For example, in the case of HER2-positive breast cancer, resistance to HER2-targeted therapies, such as trastuzumab, can occur through mutations in the HER2 receptor or the activation of alternative pathways like the PI3K/Akt/mTOR signaling cascade [81]. Furthermore, bone metastases often exhibit tumor cell heterogeneity, meaning that subpopulations of cells may possess inherent resistance traits or undergo genetic alterations that allow them to evade therapy. Immunotherapy resistance, on the other hand, can arise due to the immunosuppressive nature of the bone microenvironment, where factors like tumor-associated macrophages (TAMs), regulatory T cells, and bone-resorbing osteoclasts inhibit the immune response [82]. The upregulation of immune checkpoint proteins, such as PD-L1, in both tumor and stromal cells within the bone further prevents effective immune surveillance, undermining the efficacy of immune checkpoint inhibitors [83]. These mechanisms of resistance highlight the complexity of treating bone metastases and emphasize the need for novel approaches, such as combination therapies that target multiple pathways simultaneously, to overcome therapeutic resistance.

Thus, future research should focus on optimizing combination therapies and identifying biomarkers to predict treatment response.

### 3.3. Radiotherapy

Recent systematic reviews [84,85,86] have supported the growing role of ablative therapy and emerging techniques in managing oligometastatic disease (Table 2). Stereotactic ablative radiotherapy (SABR), also known as Stereotactic Body Radiation Therapy (SBRT), is a non-invasive procedure that delivers high doses of radiation to small, well-defined targets. In outpatient settings, it is commonly applied to brain, lung, liver, and bone metastases [87].

The efficacy of SABR across various anatomical sites has been demonstrated in multiple studies. The SABR-COMET trial [88], a landmark randomized, multicenter study, assessed patients with controlled primary tumors and 1–5 metastatic lesions. Participants were randomized to receive either palliative care or SABR. The trial reported a significant improvement in overall survival for the SABR group (41 months vs. 28 months), though a 4.5% treatment-related mortality was noted. Building on these findings, ongoing Phase III trials—SABR-COMET 10 [89] and SABR-COMET 3 [90]—are investigating the efficacy of SABR in patients with 4–10 and up to three metastatic lesions, respectively. These studies evaluate overall survival as the primary outcome, with secondary endpoints including progression-free survival, quality of life, and treatment toxicity. For prostate cancer, the STOMP and ORIOLE trials have demonstrated the benefits of SABR over observation in treating oligometastatic lesions. The STOMP trial [91], involving 62 patients with up to three metastases, showed that SABR or metastasectomy was associated with more extended androgen deprivation therapy (ADT)-free survival. However, the trial had limitations: it did not distinguish between surgical and SABR interventions, and SABR was frequently combined with supplemental ADT. The ORIOLE trial [92,93], a Phase II study, evaluated the safety and efficacy of SABR in hormone-sensitive oligometastatic prostate adenocarcinoma. SABR improved progression-free survival and reduced both 6-month progression rates and radiographic progression. Furthermore, SABR activated a systemic immune response, with baseline immune phenotypes and tumor mutation profiles showing potential as predictors of therapeutic benefit. The POPSTAR trial [94], a prospective study of 33 patients with bone oligometastases secondary to prostate cancer, explored SABR’s impact using F-NaF PET/CT imaging. At six months, significant reductions in osteoblastic activity were observed in both tumor and non-tumor bones exposed to high radiation doses. However, areas of increased uptake adjacent to treated lesions suggested that expanding clinical target volumes might be necessary. F-NaF PET has thus been proposed as a valuable tool for evaluating skeletal metastasis response [95,96]. For oligometastatic non-small cell lung cancer (NSCLC), Phase II studies by Gomez et al. [28] and Iyengar et al. [26] demonstrated that adding local consolidative radiotherapy or surgery to maintenance chemotherapy significantly extended survival compared to chemotherapy alone. Ongoing trials [97], such as NRG LU-002 and SARON, further explore the role of SBRT in NSCLC management. In pancreatic adenocarcinoma, esophageal squamous cell carcinoma, and colorectal cancer with up to five metastases, trials like EXTEND [98], ESO-Shanghai 13 [99], and ERASur [100] have shown that combining SABR or surgery with systemic therapy improves progression-free survival without increasing severe adverse events. Consistent with findings from the ORIOLE trial [92], systemic immune activation appears to be a key mechanism underlying the benefits of local treatment. The SAFRON II trial [101,102,103,104] compared single-fraction and multi-fraction SABR for pulmonary oligometastases. Since both approaches were equally practical regarding safety, systemic immunogenicity, and survival, single-fraction SABR emerged as the preferred option due to its cost-effectiveness. Finally, a review by Rubini et al. [105] explores the integration of genetic profiling into radiotherapy to enhance personalized cancer treatment. The authors discuss how identifying genetic markers can predict patients’ responses to radiation, including potential toxicities, thereby enabling tailored treatment plans that minimize adverse effects. The review also examines the impact of genetic profiling across various cancer types, highlighting its role in optimizing radiotherapy strategies and improving patient outcomes. For example, the Oncotype DX test (21 genes) and MammaPrint (70 genes) help tailor radiation therapy in breast cancer, genetic mutations in KEAP1/NFE2L2/STK11/PIK3CA are linked to radiation resistance in lung cancer, while tumor mutational burden (TMB) predicts a better response to postoperative radiotherapy in non-small cell lung cancer. Furthermore, miRNA-based biomarkers (e.g., miR-132-3p, miR-576-5p) correlate with radiosensitivity in esophageal cancer; glioblastoma genes like POLQ, PRIM1, and RPA1 are linked to radioresistance, while miR-153-3p overexpression enhances radiosensitivity. BRCA2, DAB2IP, and the DNA-PKcs inhibitor NU7441 are prostate cancer-related markers, while the methylation of ESR1 and MYOD1 and expression of SEPT9 correlate with radiotherapy response in cervical cancer.

Nevertheless, further research to develop tailored treatment plans, mainly when standard protocols are insufficient, are still needed.

### 3.4. Surgery

Surgical treatment of bone metastases, a cornerstone in managing symptomatic patients, brings significant relief and improvement, particularly in addressing SREs such as impending or established pathologic fractures, severe pain, neurological impairment from spinal metastases, and potential functional decline [18,19]. The primary objectives of surgery are to relieve pain, maintain joint function and mobility, and prevent or address pathologic fractures [106]. Recent studies prove that most patients achieve substantial pain relief after surgery, with functional improvements reported in approximately 60–80% of cases, depending on the surgical approach and disease severity [107].

The main indications for surgical intervention, such as impending or established pathologic fractures, are assessed using the Mirels scoring system [108]. This system, which evaluates lesion location, radiographic appearance size, and pain, was the first developed to determine fracture risk [109]. Surgical intervention is generally recommended for patients with high Mirels scores due to the increased risk of fracture and associated morbidity. More recently, sophisticated scoring systems based on CT scans have been proposed to improve the accuracy of fracture risk assessment and enhance surgical planning [106,110]. However, these advanced tools are not always used in clinical practice due to their complexity, leaving the Mirels scoring system as the most widely adopted in clinical settings.

Various surgical techniques have been explored, each effective in improving patient outcomes, although there is still no consensus among leading experts and surgeons in the field [111] (Table 3). A critical factor in managing bone metastases is ensuring that the implant outlasts the patient, thereby minimizing the need for revision surgeries that could delay chemotherapy and negatively impact survival outcomes [107,112,113,114,115,116,117]. Two principal surgical techniques are employed: intramedullary nailing and resection with prosthetic reconstruction. The reliability of these different surgical techniques continues to be a crucial area of investigation, as each approach presents unique benefits and risks.

Intramedullary nailing is a minimally invasive procedure to stabilize bones without extensive tissue removal. It restores weight-bearing capacity, enhances patient mobility [118], and provides immediate pain relief and quick recovery [119]. However, implant failure due to nonunion, bone loss, or disease progression may occur in long-term survivors [112,113,120]. In contrast, resection with endoprosthetic replacement is a more invasive procedure associated with higher upfront costs, increased blood loss potential, and longer recovery and rehabilitation times [121,122]. This technique, primarily indicated for metastases in the proximal femur or humerus, completely replaces the affected bone, providing more stability and reducing reoperation rates due to implant failure [113,117,121,123,124,125]. Additionally, wide resection, often part of the surgical plan for endoprosthetic replacement, can improve local control by decreasing recurrence rates and can impact prognosis [112,118,123,124,125,126,127,128,129]. Due to the complex nature of surgical planning for en bloc resection, which often requires advanced imaging and multidisciplinary collaboration, this approach is best performed in specialized centers with expertise in oncologic, orthopedic surgery. Furthermore, in the long term, it may present major complications, including infection, loosening, or dislocation [116].

Selecting the most appropriate surgical technique is crucial. A tailored approach helps optimize outcomes, minimize complications, and improve quality of life and functional recovery [112,113,114,115,116,117]. The criteria for patient stratification and optimal surgical treatment selection depend on several factors, including tumor histology, location and number of sites, extension of bone destruction, and patient’s functional status. In any case, the primary consideration is life expectancy, which can be assessed using various tools, such as PathFx [108,130,131,132]. Historically, palliative intralesional treatments, such as nailing combined with radiotherapy for better symptom management, were reserved for patients with poor prognoses [17,107,133,134,135,136,137] while aggressive treatments with curative intent, such as resection and prosthetic replacement, were recommended for patients with longer life expectancies. These included younger patients with good overall health, a long disease-free interval, favorable tumor histology, and solitary lesions [17,107,117,129,133,134,135,136,137].

In recent years, treatment strategies have been redefined with the introduction of the oligometastasis concept, which suggests that patients with a limited number of metastases may also benefit from aggressive interventions, including surgery [21,22,28,138]. Although the precise role of surgical resection in oligometastatic disease remains under investigation, emerging evidence indicates favorable outcomes. Soran et al. [139], in a multicenter prospective study, evaluated luminal A/B and HER2-positive breast cancer patients with up to five operable metastases in the lungs, liver, or bones after primary cancer treatment. The study found a significantly lower risk of death in patients who underwent surgical excision of metastases compared to those who received systemic therapy alone. Similarly, Ferriero et al. [140] analyzed patients with renal cell carcinoma and controlled primary tumors with up to three metastases. Their findings demonstrated superior overall survival (OS) in patients treated with metastasectomy and systemic therapy compared to systemic therapy alone, even with long-term follow-up. Specifically, 2-year, 5-year, and 10-year OS rates were 93.8%, 82.8%, and 79.5%, respectively, for the combined approach, compared to 70.5%, 52.9%, and 41.9% for systemic therapy alone (*p* < 0.001). With regard to bone metastases, several studies report no significant differences in survival between patients with resected oligometastatic lesions and those with solitary lesions, highlighting the potential benefits of surgical resection in major bones for oligometastatic patients [24,118,128,141]. These findings reinforce the role of aggressive surgical management as a viable option to improve survival and quality of life in this patient population.

While aggressive surgical interventions have shown promise for oligometastatic patients, specific lesions and anatomical sites may not require extensive resection. Minor lesions in areas such as the ribs, clavicle, and distal ulna often respond well to radiotherapy or chemotherapy alone, making surgical intervention unnecessary [129]. Moreover, limited surgical approaches must be considered, particularly for metastases in the pelvis and spine [142,143,144], where the risk of severe postoperative complications, like infections or neurological impairment, is significantly higher. Minimally invasive strategies such as percutaneous vertebroplasty, kyphoplasty, and radiofrequency ablation (RFA) are increasingly used to manage spinal and pelvic metastases. For pelvic metastases, minimally invasive options like percutaneous cementation and nailing are often recommended [142,145]. For example, radiofrequency ablation has effectively palliated pain in metastatic spinal lesions. Combined with cement augmentation, it reduces pain and reinforces bone stability [142,143]. Similarly, electrochemotherapy has emerged as an effective, less invasive alternative for spinal metastases [144]. Nevertheless, these procedures address symptoms and do not eliminate the tumor burden; thus, minimally invasive techniques need to be combined with systemic therapies and radiation to maximize patient outcomes by balancing symptom management with disease control.

The oligometastatic paradigm is undoubtedly appealing to surgeons and patients. In conclusion, it is essential to remember that to attempt a healing purpose, all oligometastases must be treated, not just one. Consequently, multidisciplinarity and collaboration between surgeons, radiotherapists, and oncologists is pivotal.

### 3.5. Multidisciplinarity

Since the primary goal of managing metastatic patients is to achieve disease control or prolong survival while preserving quality of life, an interdisciplinary approach involving specialists from various fields is essential. Key members of the care team typically include oncologists, orthopedic surgeons, radiologists, radiotherapists, pathologists, and pain management specialists, each contributing to treatment selection based on tumor biology, disease burden, and patient prognosis.

Prognostic models help estimate survival and guide treatment intensity, while biomarkers aid in identifying patients who may benefit from local therapies. Errani et al. [146] highlight the importance of prognostic models in determining optimal treatment for metastatic bone disease, where life expectancy plays a crucial role in decision-making. Commonly used tools include PathFx, Optimodel, SPRING, and the IOR score. PathFx is particularly useful for short-term survival predictions (3- and 6-month outcomes), especially in surgical decision-making, while Optimodel has shown the highest accuracy for 12- and 24-month survival estimates. Barnum and Weiss et al. [147] categorize biomarkers into tumor-based, blood-based, and imaging-based types. Tumor-based biomarkers, derived from tumor tissue, provide insights into oligometastatic behavior, with genetic mutations (e.g., KRAS, BRAF, TP53, and SMAD4) and microRNAs (e.g., the miR-200 family and 14q32 miRNAs) linked to prognosis and treatment response. Blood-based biomarkers, such as circulating tumor DNA (TP53 mutations), circulating tumor cells, and serum markers (e.g., LDH and ALP), offer real-time monitoring of disease status, recurrence risk, and therapy response. Imaging-based biomarkers, including PSMA PET/CT for prostate cancer and radiomic analysis, improve oligometastatic disease detection and characterization. Given the complexity of interpreting these parameters, different experts are required to reach the most appropriate treatment for every patient.

Beyond survival, the quality of life (QoL) in patients with skeletal metastases must be considered. It can vary depending on tumor histotype, extension of bone involvement, overall health status, and treatment impact. Indeed, while local and systemic therapies can prolong survival or offer curative potential, they may also impair physical function and well-being due to side effects. Surgery, like intramedullary nailing and endoprosthetic replacement, offers significant benefits even if it may reduce mobility and fatigue during the initial period or require extended rehabilitation, especially in prosthetic reconstructions. Palliative radiotherapy has been shown to effectively manage symptomatic bone metastases, providing a high degree of pain control and improving QoL, but it is not effective in tumor progression control [148]. On the other hand, advanced techniques, like stereotactic body radiotherapy (SBRT), offer precise targeting of metastatic lesions, potentially enhancing local control even if not significantly improving pain response or QoL compared to conventional radiotherapy [149]. Chemotherapy is often associated with fatigue, nausea, and bone marrow suppression; immunotherapy may cause immune-related adverse consequences, whereas target therapies generally have fewer side effects. In this scenario, palliative care has a central role in managing pain, improving function, and enhancing comfort for patients with extensive skeletal involvement. Non-invasive alternatives for pain palliation in bone metastases, such as high-intensity focused ultrasound (HIFU), can be an alternative to traditional treatments for managing pain related to bone metastasis. Bongiovanni et al. [150] investigated the application of 3-Tesla magnetic resonance-guided high-intensity focused ultrasound (3 T-MR-HIFU) to alleviate pain caused by bone metastases from solid tumors. Their study demonstrated that 3 T-MR-HIFU is a promising non-invasive treatment option, providing significant pain relief. Similarly, Bertrand et al. [151] reviewed the effectiveness and feasibility of focused ultrasound for treating bone metastases, highlighting its potential as a non-invasive therapeutic approach.

Despite the availability of advanced treatments, it is also mandatory to consider patient perspectives, which often include concerns about the long-term effectiveness of pain management options, as well as the need for ongoing emotional and psychological support during the progression of their illness. Adherence to prescribed treatments is another significant challenge for patients with bone metastases, as factors such as pain, side effects, and the complexity of the treatment regimen can affect a patient’s ability or willingness to follow through with therapy. Patients may struggle with the frequency of medication or therapy sessions, especially when managing multiple medications or appointments across various specialties. Financial burdens also play a significant role in treatment adherence, as the costs associated with ongoing cancer care, including medications, travel to treatment centers, and hospitalization can be overwhelming for many patients. Even with insurance coverage, out-of-pocket costs can be high, creating barriers to the most effective treatments. Additionally, patients may face challenges related to insurance limitations, lack of coverage for newer therapies, or financial insecurity, leading to reduced treatment adherence and negatively impacting their overall health outcomes. Therefore, personalized care plans that balance survival benefits with QoL considerations are essential.

Guidelines from the National Comprehensive Cancer Network (NCCN) and the European Society for Medical Oncology (ESMO) [152,153,154,155,156] emphasize the importance of multidisciplinary approaches, such as tumor boards, to evaluate diagnostic findings and tailor local and systemic treatments based on the latest evidence and patient preferences. These approaches have improved clinical outcomes, enhanced adherence to care standards, and supported more balanced treatment decisions. Multidisciplinary management is precious for complex cases, such as skeletal metastases, where clear guidelines may be lacking. It ensures that surgery is effectively integrated and coordinated with systemic therapies and radiation, tailoring treatment to the patient’s disease stage and clinical condition to optimize outcomes. Additionally, comprehensive patient care is crucial to address physical, psychological, and palliative needs throughout the disease trajectory. Finally, tumor boards are critical in defining oligometastatic disease and identifying candidates for aggressive treatments. Single-center studies by Christ et al. [157] and Galata et al. [158] reported higher rates of local treatment (47% and 68%, respectively) in patients reviewed by multidisciplinary teams. Similarly, Choi et al. [29], in a prospective trial on colorectal cancer patients with oligometastases, observed an 89.9% rate of local treatment in patients managed within a multidisciplinary setting, achieving a five-year survival rate of 74%, compared to a 48% in patients who did not receive local treatment. Other studies [159,160] have also demonstrated improved one- and five-year survival rates in patients managed through multidisciplinary approaches. Notably, Choi et al. [161] and Lee et al. [162] reported better survival outcomes even in patients with more than five metastatic lesions, supporting the expansion of aggressive treatment criteria and highlighting the need for further research. Indeed, specific recommendations for selecting patients who would benefit the most from such approaches are still needed.

Multidisciplinary treatment allows for early oligometastatic status assessment, prompt identification and staging, which are crucial for guiding treatment decisions. This proactive approach ensures that interventions are tailored to individual patient factors, probably improving outcomes.

However, the multidisciplinary approach has some limitations.

First, despite an effective communication among specialists is crucial, it is often tricky, leading to delays in starting treatment, fragmentation of patient management, and ultimately, reduced survival [163]. Indeed, different clinical perspectives may result in disagreements as specialists from different fields prioritize different aspects of care. For example, while surgical oncologists may focus on resecting isolated metastases, radiation oncologists might advocate for SBRT, and medical oncologists may favor systemic therapies like chemotherapy, targeted treatments, or immunotherapy. Additionally, the vast amount of medical information and complex treatment plans can overwhelm patients, increasing psychological distress and reducing adherence to therapy [164]. Another challenge is the variability in expertise among tumor board members, particularly regarding newer treatments such as immune checkpoint inhibitors or novel targeted therapies, which may lead to inconsistencies in recommendations. Finally, even though some studies suggest a survival benefit [157,158,161,162], the evidence is inconsistent across all cancer types and settings.

In a recent paper, Smith et al. [165] evaluated the impact of multidisciplinary team (MDT) meetings in surgical oncology by analyzing their influence on patient management decisions. They reviewed 438 cases across 30 MDT meetings. Findings showed that MDT discussions altered treatment plans for nearly 50% of patients, with 89.62% of MDT recommendations being implemented. However, the predictability of recommendations was inconsistent, and no clear patient factors, except for the female sex, were associated with correct prediction.

Thus, further studies are needed to understand better which cases a multidisciplinary approach would benefit.

## 4. Conclusions and Future Directions

The surgical management of bone metastases presents a unique set of opportunities and challenges in advancing cancer care. Surgical interventions have become essential to improving quality of life and function, especially in patients with oligometastatic disease, where targeted, more aggressive treatment (resection and prosthetic replacement) may offer prolonged survival and better functional outcomes. It is mandatory to incorporate surgery into a multidisciplinary treatment strategy that includes systemic and radiation therapies to optimize treatment timing, enhance therapeutic effectiveness, and ease patient care.

However, significant gaps in research still need to be filled. The ideal timing for surgical intervention (Should surgery precede or follow systemic treatment?) needs to be defined. Moreover, current scoring systems, like Mirels, provide a simplistic picture; more comprehensive predictive models that account for individual patient variables and cancer biology are needed.

## Figures and Tables

**Table 1:** Summary of systemic treatment for bone metastases.

Therapy Type	Mechanism of Action	Indications	Advantages	Limitations
*Bone-Modifying Agents*	Inhibit osteoclast activity and bone resorption	Multiple cancers (breast, prostate, lung, myeloma)	Reduce skeletal-related events, improve quality of life	Renal toxicity (bisphosphonates), risk of osteonecrosis of the jaw
*Hormone* *Therapy*	Blocks hormone-driven tumor growth	Hormone-sensitive cancers	Targets tumor-specific pathways, fewer systemic side effects	Resistance development, side effects like osteoporosis, fatigue
*Targeted* *Therapy*	Inhibits cancer-specific molecular pathways	Cancers with specific genetic mutations	Precision therapy, fewer off-target effects	Requires biomarker testing, resistance can develop
*Immunotherapy*	Enhances immune response against cancer cells	Renal cell carcinoma, NSCLC, melanoma with bone metastases	Durable response in some patients, potential for long-term remission	Limited efficacy in bone metastases due to bone microenvironment
*CAR-T Therapy*	T cells genetically modified to attack tumor cells	Multiple myeloma with bone involvement	Highly specific, promising results in hematologic malignancies	Requires specialized centers, high cost, potential severe immune-related toxicities

**Table 2:** Summary of radiotherapic treatments for bone metastases.

Therapy Type	Mechanism of Action	Indications	Advantages	Limitations
*Conventional * *Radiotherapy*	Lower radiation doses over multiple sessions	Palliation of bone pain, prevention of fractures in advanced disease	Pain relief, prevents disease progression	Less effective for oligometastatic disease, may require multiple sessions
*Stereotactic Ablative Radiotherapy (SABR/SBRT)*	High-dose radiation to a small, precise area	Oligometastatic disease, bone metastases in controlled primary tumors	Improves survival, systemic immune activation	Requires specialized equipment, potential treatment-related toxicity
*Radiopharmaceuticals*	Radium-223	Bone-targeting radioactive agents for widespread bone metastases	Selectively targets bone metastases with minimal damage to normal tissue	High cost, selected tumor types (e.g., prostate cancer)
*Genetic Profiling in Radiotherapy*	Biomarker-based approach (Oncotype DX, KEAP1/NFE2L2 mutations, TMB levels)	Predicts radiosensitivity	Tailors radiotherapy for better outcomes	Requires advanced testing, not widely available

**Table 3:** Summary of surgical treatments for bone metastases.

Therapy Type	Mechanism of Action	Indications	Advantages	Limitations
*Intramedullary Nailing*	Stabilization without tumor removal	Impending or established pathological fractures (e.g., long bones)	Minimally invasive, preserves function, rapid recovery	Risk of implant failure in long-term survivors
*Resection with Endoprosthetic Reconstruction*	Bone removal + prosthetic replacement	Single lesion Oligometastatic disease Large metastases in weight-bearing bones (proximal femur, humerus)	Durable, reduces reoperation rates, improves local control	Higher cost, longer recovery, potential complications (infection, dislocation)
*Minimally * *Invasive * *Techniques*	Cement augmentation or tumor ablation	Spinal, pelvic, or non-weight-bearing bone metastases	Reduced morbidity, pain relief, shorter hospital stay	Does not eliminate tumor burden, requires combination with systemic therapy
